# Confronting the TB‐HIV Syndemic in Adolescents and Young Adults: A Call to Action in a Time of Crisis

**DOI:** 10.1002/jia2.70100

**Published:** 2026-03-23

**Authors:** Leslie A. Enane, Adam Leonard, Lameck Diero, Olivier Marcy, Marcel Yotebieng

**Affiliations:** ^1^ The Ryan White Center for Pediatric Infectious Disease and Global Health Department of Pediatrics Indiana University School of Medicine Indianapolis Indiana USA; ^2^ Indiana University Center for Global Health Indianapolis Indiana USA; ^3^ Center for Infectious Disease and Nursing Innovation Johns Hopkins School of Nursing Baltimore Maryland USA; ^4^ Center for Global Nursing University of California San Francisco Institute for Global Health Sciences San Francisco California USA; ^5^ Department of Medicine Moi University College of Health Sciences Eldoret Kenya; ^6^ University of Bordeaux Inserm U1219 Bordeaux Population Health IRD EMR271 GHiGS Bordeaux France; ^7^ Division of General Internal Medicine Department of Medicine Albert Einstein College of Medicine Bronx New York USA

1

Tuberculosis (TB), as the leading infectious cause of death globally and the most common opportunistic infection among people with HIV, imposes a profound burden on adolescents and young adults (AYA, ages 10–24 years) [1]. Although preventable and curable, TB remains a leading cause of death for AYA in endemic settings, the predominant cause of morbidity and mortality in AYA living with HIV (AYALHIV)—even in the era of antiretroviral therapy (ART)—and an increasingly recognized driver of long‐term disability and diminished quality of life [2, 3]. Developing TB during adolescence or young adulthood may impact future health, educational attainment, livelihoods, and mental and social wellbeing [2]. Approximately 2 million AYA developed TB in 2024, representing ∼18% of all people who fell ill with TB (Figure 1). Despite their TB burden, AYA remain underrepresented in surveillance, clinical trials and programmatic priorities, and critical gaps in prevention, treatment and post‐TB care for this vulnerable group remain unaddressed.

**FIGURE 1:**
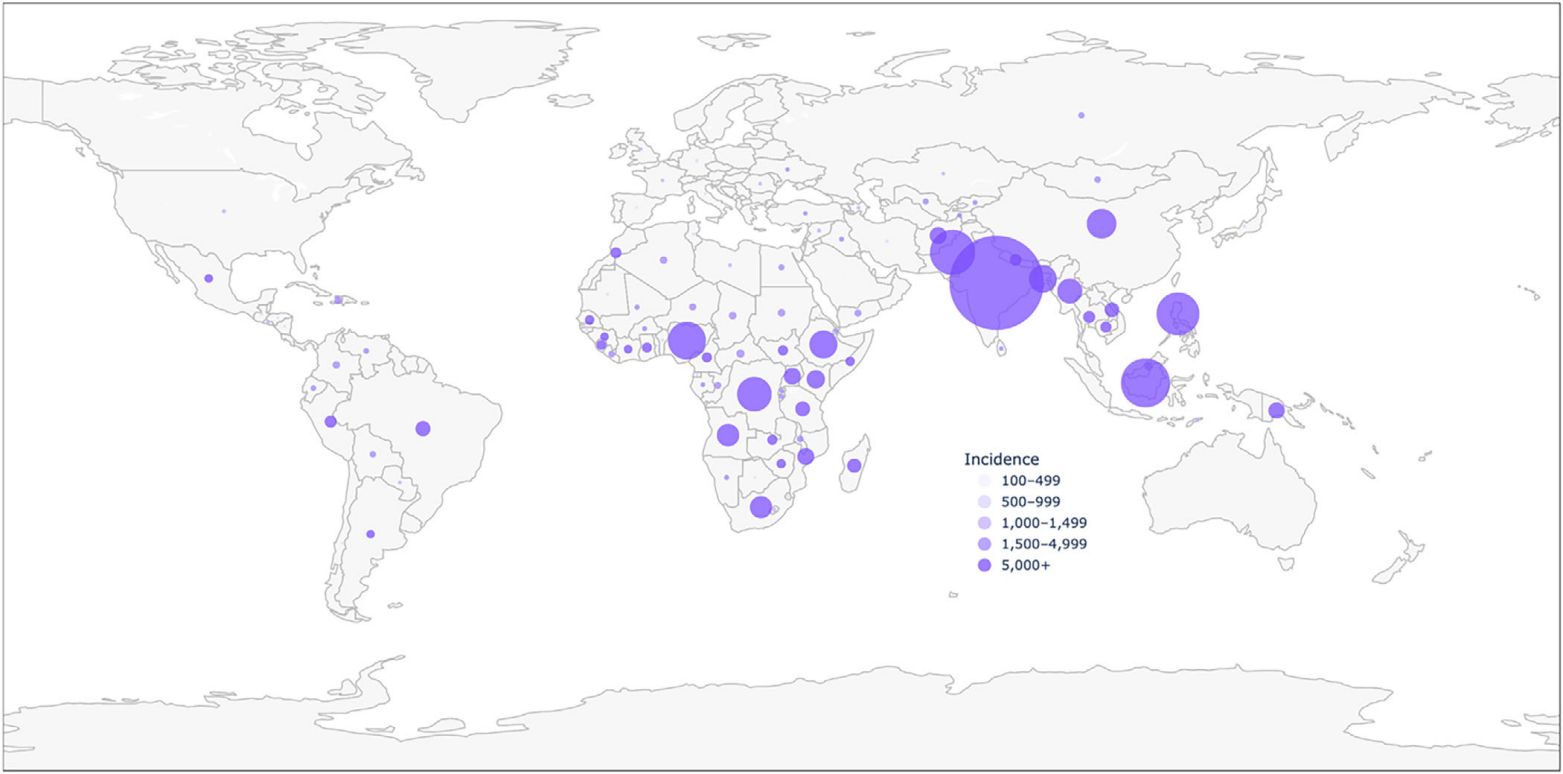
Estimated TB incidence in adolescents and youth ages 10–24, in 2024. Map developed from World Health Organization (WHO) TB incidence estimates disaggregated by age [1]. Data available at https://www.who.int/teams/global‐programme‐on‐tuberculosis‐and‐lung‐health/data.

Current literature on TB in AYA is limited; however, it is clear that AYA face distinct vulnerabilities to TB and challenges in TB prevention and treatment [2]. Physiologic changes in adolescence drive markedly increased risk for progression to active TB and development of adult‐type cavitary disease [4]. Further developmental, behavioural and environmental factors impacting TB risk in AYA include high mobility, congregate environments (such as schools and dormitories), malnutrition (related to ongoing growth and food insecurity), air pollution (given its impact on lung development) and substance use. Once established, TB diagnosis is frequently delayed, resulting in ongoing transmission and advanced illness [3]. TB treatment in AYA is complicated by stigma and mental health burdens, adherence challenges and greater loss to follow‐up (LTFU) from treatment than in other age groups [3, 5, 6, 7, 8, 9].

Among AYA, those living with HIV face the greatest TB risk, driven by immunosuppression, gaps in ART, and convergent developmental and social barriers to care engagement. Although TB preventive therapy (TPT) is recommended for all people with HIV in TB‐endemic settings [10, 11], AYALHIV experience low TPT uptake and completion. A recent study in Kenya found that only 42% of AYALHIV initiated TPT, and of those with ≥6 months of follow‐up, just 54% completed it—representing a critical missed opportunity [10]. When TB does occur, management may be complicated by severe or disseminated disease presentation, pill burden and drug interactions, as well as the psychosocial burdens of compounded isolation and stigma. Ultimately, AYALHIV face higher LTFU from TB treatment and TB mortality than their HIV‐negative peers [5, 6, 7, 8].

Even after successful treatment, emerging data show adolescents experience prevalent post‐TB lung disease (PTLD). Studies have found that 57–65% of adolescents have impaired lung function after TB treatment, and comparatively worse lung function in adolescent TB survivors than in adolescent contacts without TB [12, 13, 14]. Further data are needed to evaluate PTLD among AYALHIV, who have been found to have worse lung function than adults with HIV, with previous TB being a risk factor [15].

Despite years of progress against HIV and TB, AYA continue to fall through the cracks of global health systems not designed to meet their needs. Recent recognition of the neglect of TB in AYA has spurred emerging research and policy efforts. However, we now confront acute threats that have made AYA even more vulnerable. The COVID‐19 pandemic set back already slow progress in TB control, driving increases in TB illness and mortality. Now, a global health funding crisis fundamentally threatens the TB and HIV response. We cannot accept the resulting avoidable illnesses and deaths for AYA and other vulnerable populations.

Addressing TB as a leading cause of death in AYA demands urgent, dedicated action. Global TB policies have begun to recognize adolescent needs and incorporate age‐stratified surveillance, yet significant implementation gaps call for greater attention to this group [2, 16]. We outline key areas that should be addressed through focused strategic priorities, programming, interventions and research.

Implementation of AYA‐friendly, high‐quality, integrated TB and HIV services has been shown to improve outcomes for AYALHIV [17]. In Kenya, TPT initiation for AYALHIV was higher in clinics with lower patient‐to‐staff ratios or designated AYA areas [10]. Adolescent‐friendly health centres may improve access to TB and HIV care within comprehensive service delivery. However, while WHO guidance emphasizes accessible, acceptable, equitable, appropriate and effective care, adolescent‐friendly models of TB care that are implemented in partnership with AYA are lacking [2, 18]. Evidence‐based interventions are needed to increase TB treatment adherence and completion among AYA. Community‐based treatment support, peer interventions, digital tools and financial interventions may be adapted for youth with TB. In Uganda, the 99DOTS digital adherence intervention increased treatment success in AYA, and decreased LTFU [19].

Research agendas should identify, evaluate and scale up evidence‐based interventions for AYA with TB or TB‐HIV. This includes research in case‐finding strategies; diagnostics; treatment and prevention regimens, including for drug‐resistant TB; PTLD prevention and management; vaccines; and implementation science that identifies what works in real‐world AYA environments. Investment in longitudinal cohorts and routine data systems that capture AYA‐specific outcomes is critical for accountability and impact. Addressing the structural and psychosocial vulnerabilities that impact AYA treatment outcomes must also be a priority. Interventions for stigma reduction and integrated psychosocial support are needed. Malnutrition, food insecurity and catastrophic costs of TB must be addressed to support recovery. Funding and research to translate advances into accessible services for youth will help ensure benefits reach the most vulnerable.

Intentional and meaningful partnership with youth with lived experience of TB and HIV is essential for co‐designing and implementing AYA‐responsive services and upholding rights‐based care, and is integral to the success of national and programmatic goals in ending TB. Examples include establishing advisory boards of AYA with lived experience of TB and HIV—an approach our team has implemented across five African countries in the Youth TB Sentinel Research Network study. Youth engagement and leadership will help ensure that research and interventions are grounded in the lived realities and priorities of young people.

AYA bear disproportionate and preventable burdens from TB and TB‐HIV, yet remain underserved globally. We have clear paths forward to close implementation gaps; advance AYA‐friendly, integrated TB and HIV services; support treatment completion and recovery; and invest in research and evidence‐based programming—with youth at the forefront of our response. We must advocate for funders and governments to invest in ending TB and HIV to secure a healthy future for young people.

## Author Contributions

LAE conceived and led the writing of this manuscript. All authors contributed to the revision of the manuscript and have approved the final version.

## Conflicts of Interest

The authors have no conflicts of interest to declare.

## Funding

Research conducted by Enane is supported by the National Institute of Allergy and Infectious Diseases of the US National Institutes of Health, under Award Numbers R01AI184174 and U01AI69911. Research conducted by Leonard is supported by the National Institute of Nursing Research of the National Institutes of Health under Award Number T32NR020315. Enane's research is additionally supported by the Indiana University Center for Global Health, the Arnhold Institute for Global Health at Icahn School of Medicine at Mount Sinai in partnership with Moi University and Moi Teaching and Referral Hospital, Indiana University Dance Marathon, and Riley Children's Foundation.

## Disclaimer

The content is solely the responsibility of the authors and does not necessarily represent the official views of the National Institutes of Health or of the other funders.

## Data Availability

The data presented in this viewpoint article are from WHO Global tuberculosis report 2025 and are available at https://www.who.int/teams/global‐programme‐on‐tuberculosis‐and‐lung‐health/data.
